# Genomic Adaptation, Environmental Challenges, and Sustainable Yak Husbandry in High-Altitude Pastoral Systems

**DOI:** 10.3390/vetsci12080714

**Published:** 2025-07-29

**Authors:** Saima Naz, Ahmad Manan Mustafa Chatha, Qudrat Ullah, Muhammad Farooq, Tariq Jamil, Raja Danish Muner, Azka Kiran

**Affiliations:** 1 Department of Zoology, Government Sadiq College Women University, Bahawalpur 63100, Pakistan; azkakiran.z@gmail.com; 2 Department of Entomology, Faculty of Agriculture and Environment, The Islamia University of Bahawalpur, Bahawalpur 63100, Pakistan; manan.chatha@iub.edu.pk; 3 Department of Theriogenology, Cholistan University of Veterinary and Animal Sciences, Bahawalpur 63100, Pakistan; 4 Department of Soil Plant and Food Science, University of Bari Aldo Moro, Via Giovanni Amendola, 165/a, 70121 Bari, Italy; muhammad.farooq@uniba.it; 5 Independent Researcher, 07743 Jena, Germany; drtariqjamil01@gmail.com; 6 Department of Animal Breeding and Genetics, The University of Agriculture, Dera Ismail Khan 29111, Pakistan; danishmunirraja@gmail.com

**Keywords:** yak (*Bos grunniens*), genomics, hypoxia adaptation, climate changes, genetic improvement

## Abstract

The yak (*Bos grunniens*) is a key species in high-altitude rangelands of Asia. Despite their ecological and economic importance, yak production faces persistent challenges, including low milk yields, vulnerability to climate changes, emerging diseases, and a lack of systematic breeding programs. This review presents the genomic, physiological, and environmental dimensions of yak biology and husbandry. Genes such as *EPAS1*, which encodes hypoxia-inducible transcription factors, underpin physiological adaptations, including enlarged cardiopulmonary structures, elevated erythrocyte concentrations, and specialized thermoregulatory mechanisms that enable their survival at elevations of 3000 m and above. Copy number variations (CNVs) and single nucleotide polymorphisms (SNPs) present promising markers for improving milk and meat production, disease resistance, and metabolic efficiency. F1 and F2 generations of yak–cattle hybrids show superior growth and milk yields, but reproductive barriers, such as natural mating or artificial insemination, and environmental factors limit the success of these hybrids beyond second generation. Infectious diseases, such as bovine viral diarrhea and antimicrobial-resistant and biofilm-forming *Enterococcus* and *E. coli*, pose risks to herd health and food safety. Rising ambient temperatures, declining forage biomass, and increased disease prevalence due to climate changes risk yak economic performance and welfare. Addressing these challenges by nutritional, environmental, and genetic interventions will safeguard yak pastoralism. This review describes the genes associated with different yak traits and provides an overview of the genetic adaptations of yaks (*Bos grunniens*) to environmental stresses at high altitudes and emphasizes the need for conservation and improvement strategies for sustainable husbandry of these yaks.

## Simple Summary

This manuscript overviews the biology, genetics, and husbandry practices of yaks (*Bos grunniens*) inhabiting high-altitude Asian rangelands. The genomic adaptations to hypoxia, the implications of climate change, and the risk of emerging diseases on yak production are discussed. Moreover, breed distribution, production traits, and crossbreeding of yak–cattles is reviewed. An emphasis is placed on sustainable breeding, disease surveillance, and climate-based husbandry practices for safeguarding the livelihood of the pastoral communities in these ecosystems.

## 1. Introduction

The yak (*Bos grunniens*) is a remarkable mammalian species that inhabits the high-altitude rangelands of Asia, which extend from the Hengduan Mountains of China; continue through the Shimshal valley in the Karakoram mountains of Pakistan; span across the Pamir mountains of Tajikistan and Afghanistan; and part into Russia, Mongolia, and Kyrgyzstan [1,2]. Yaks are adapted to extreme environmental conditions where annual the average temperature can range between 45 °C in summer and −20 °C in winter, at elevations of an average of 3500 m, depending upon location and altitude [3,4]. They are vital sources of milk, meat, transportation, hair, draught power, and fuel for the local people. Everyday life and sociocultural customs of Tibetans and other local groups are strongly influenced by yaks; hence, they are revered and seen as “wealth” by the local population [5]. In the areas where vegetation is scarce, yak herding forms the backbone of subsistence and cultural practices [4].

Beyond their economic roles, yaks possess unique biological characteristics that enable them to thrive in harsh climates, hypoxic conditions, and UV-intensive environments, efficiently converting scarce vegetation into high-quality nutrients; thus, they are also essential for preserving the region’s biodiversity and gene pool [6,7]. In their natural habitat, yaks co-exist with their native species; however, little is known about their precise timing of domestication [8]. In order to stay healthy in the oxygen-poor environment, Tibetan nomads rely on yak milk and its derivatives in their daily diets. Yak milk has higher protein, specific enzymes, antioxidants, vitamins, and essential fatty acids [9]. Despite this, commercial production of yak milk is restricted by lower yields in traditional systems, typically 150–500 kg per lactation [10]. Therefore, enhancing milk yields is a significant goal in yak husbandry. The importance of yaks extends to Tibetan and other peoples’ religion, mythology, and traditional medicine [5,11]. For example, yak blood, drawn from young animals is believed to have therapeutic effects and is administered to people suffering physical weakness and jaundice in Nepal’s Mustang region [12]. The ingredients extracted from yaks are believed to possess mystical properties [11].

However, yak herding is faced by multiple challenges, such as poverty, environmental degradation, and climate change, which have disrupted traditional pastoral and migration patterns, resulting in fragmentation of the pastures herders had relied upon for generations [5]. These factors have pushed yak herders to a socioeconomic vulnerability, calling for yak production and sustainability as an urgent priority. Therefore, recent research has increasingly focused on the genetic and molecular mechanisms that adapt yaks to the harsh environments of high-altitude habitats. According to previous research, the heterozygosity rate of yaks is around 1.5 times greater than that of cattle (*Bos taurus*), and yak-specific gene families are overrepresented in regions linked to immunology, host defense, and olfactory perception [6]. Yaks also have >590 gene families, related to energy metabolism and sensory perception. Positive selection is also evident in genes related to energy consumption and hypoxia adaption [6]. Mitochondrial gene expression patterns have evolved to reduce respiratory chain activity, thereby reducing overall energy demands in skeletal muscles and better adaptability in lower oxygen conditions [13].

These unique genetic adaptations provide a valuable model for understanding mammalian resilience in extreme environments and also offer practical implications to improve yak production and the health of yaks through informed breeding strategies [13,14]. Increasing the genetic potential for higher milk and meat yields, improved disease resistance, and higher metabolic efficiency are of both scientific and practical importance, thus conserving the biodiversity and strengthening and supporting the sustainability of the livelihoods of yak herders. By integrating molecular genetics, traditional knowledge, and modern husbandry practices, the future of this iconic species and dependent communities can be secured. This review highlights the genes associated with various yak traits and provides an overview of the genetic adaptations of yaks to environmental stressors in high-altitude ecosystems.

## 2. Genomic Insights into Hypoxia Tolerance in Yaks

High altitudes can impair biological functions even in animals well-adapted to such environments. Previously, high-altitude environmental exposure, such as low temperatures and hypobaric hypoxia, effected the human immune system and increased susceptibility to infections, skeletal deterioration, autoimmune disorders, and cancer [15,16]. High-altitude exposure impaired the liver functions of and heightened the oxidative stress in mice [17]. Chronic hypoxia and oxidative stress also affected the fertility and reproductive traits of sheep, leading to impaired corpus luteum formation and function and reduced intrauterine growth [18,19,20]. These native high-altitude populations of *B. grunniens* have successfully adapted themselves to chronic hypoxia at high elevations over many generations, despite belonging to the genus *Bos*, which is closely related to domestic cattle [14,21]. Additionally, yaks possess longer, wider, and rounder pulmonary–artery endothelial cells with minimal smooth muscle, enabling superior performance in high-altitude environments compared to cattle [22,23].

The harsh conditions at high altitudes are exacerbated by the low oxygen levels, frigid temperatures, and limited forage availability [24]. Yaks typically inhabit alpine regions that are between 3000 and 6000 m, where frost occurs year-round. Yaks have evolved thick outer coats without functional sweat glands, which reduces heat loss and enhances cold resistance [14]. Moreover, a thick fleece, consisting of an exterior layer of long coarse hair, which grows before winter, and a lush undercoat of fine down fibers, which appears in winter, covers the entire body, retaining body heat and repelling moisture [1,14,25]. Compared to domestic cattle, yaks have a thicker keratinized epithelium, larger and more frequent conical papillae, and shorter tongues with greater lingual prominence [26].

Gene investigation studies have identified a number of genes associated with high-altitude adaptation in yak populations. These genes are mainly associated with physiological processes that react to hypoxia, temperature acclimation, changes in the cardiovascular system, and energy metabolism [14,27]. The most promising candidate gene for the hypoxia-inducible transcription factor (HIF-2α) is endothelial PAS domain-containing protein 1 (EPAS1). EPAS1 is thought to control the production of erythropoietin, which varies depending on the amount of oxygen present in the cellular environment at high altitudes [28,29].

**Figure 1:**
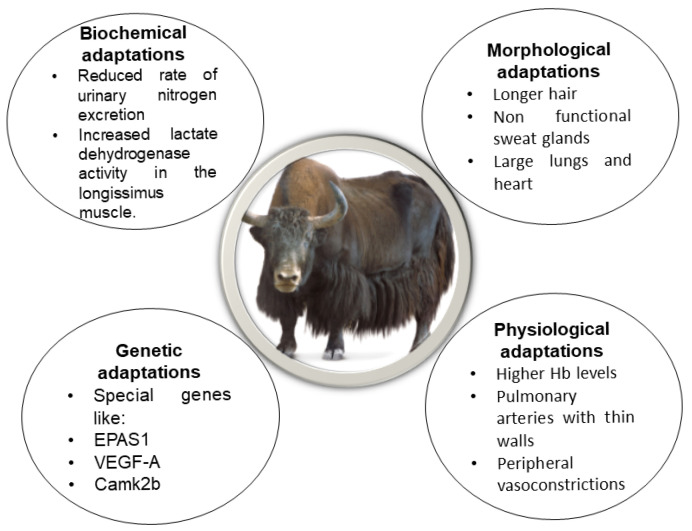
High-altitude adaptations in yaks.

Yaks, especially those inhabiting the Qinghai–Tibetan Plateau, have evolved unique physiological adaptations that allow them to survive under harsh conditions. Compared to other cattle species, yaks possess larger hearts and lungs, thinner alveolar septa and blood–air barriers, and a greater pulmonary alveolar surface area. They also have higher concentrations of hemoglobin and erythrocytes in their blood, enabling them to flourish in low-oxygen environments [6,13]. Yaks also exhibit thin-walled pulmonary arteries with minimal smooth muscle and lack right ventricular hypertrophy—further supporting adaptations to hypoxia [30]. Together, these cardiovascular characteristics help yaks to function efficiently under chronic hypobaric conditions [31]. These adaptations improve hypoxic pulmonary vasoconstriction without inducing excessive hemoglobin or red blood cell production and likely are the outcome of natural selection [31]. To better understand these evolutionary adaptations, this review presents the genes linked to key production traits, such as milk and meat yields. These findings underscore the importance of integrating genomic data with ecological and phenotypic insights to unravel the complex mechanisms underlying adaptations in high-altitude yaks. Adaptations of yaks to high altitudes are summarized in Figure 1.

## 3. Genetic Resources of Yaks

Genomic selection techniques in yak breeding have accelerated genetic improvements by identifying diverse genomic variations, including variable numbers of tandem repeats; single nucleotide polymorphisms (SNPs); structural changes, such as deletions; and transposable elements. Although SNPs were traditionally considered as the primary source of genome variation, copy number variations (CNVs) have recently gained recognition as significant markers for assessing genetic diversity and evolutionary patterns. Due to their greater nucleotide content compared to SNPs, CNVs hold promising potential for evaluating these genetic attributes [32]. The Gannan yak is one of 18 distinct genetic resources in China and is known for its adaptability, high nutritional value, and pleasant meat taste [33,34]. To develop Kecai yaks, wild yaks were crossed with Gannan yaks over several generations. Kecai yaks, mainly raised in Kecai Town of Xiahe County in Gansu Province, are distinguished by their black coats, horns, and fluffy hair on the chest and under the tail. They are valued due to their large size, their high fecundity, and the high hereditary link of the taste of their meat with the Gannan yak [35]. However, little is known about their genetic characteristics, population structure, and unique traits, warranting further investigations.

Over time, species have evolved unique traits to survive extreme terrestrial environments through natural selection [36]. Genetic variation within yak populations is essential for survival, adaptability, and resilience to environmental changes [37,38]. Traditionally, population genetics emphasized structural sequence variations, often treating individual genes as independent evolutionary units [39]. However, many adaptive traits are shaped by polygenic mutations, making their genetic signals more challenging to detect through conventional selection scanning methods, which are typically normalized for identifying selective sweeps at a single loci [40]. Adaptive evolution at the genetic level can occur either through selective sweeps involving a few loci with large phenotypic effects or through simultaneous changes in allele frequencies across numerous loci with smaller individual effects [40]. Recent advancements in high-throughput sequencing and genotyping technologies have facilitated identification of population-specific genome signatures, particularly those linked to livestock adaptation to environmental stressors. For yak populations, several genes have been identified as potentially contributing to high-altitude adaptations. These genes are primarily associated with physiological processes that mitigate hypoxia and thermoregulation and modulate cardiovascular function and energy metabolism [31,41].

## 4. Available Breeds of Yaks

Once, the estimated wild yak population on the Tibetan plateau was more than one million, which significantly decreased to about 10,000 animals due to human poaching, habitat destruction, and genetic dilution through interbreeding with cows [42]. Domestic yaks are bred locally in Pakistan, Nepal, China, India, Kyrgyzstan, Russia, Tajikistan, Afghanistan, Bhutan, and Mongolia. These nations are broadly divided into northern and southern yak-rearing regions, connected in the west by the Pamir Mountains [5]. As of 2003, there were an estimated 14.2 million yaks (*B. grunniens*) on the globe, the majority of which concentrated in China and Mongolia, and smaller populations distributed in the Himalayan belt and Central Asia [43]. China has identified at least 12 distinct yak breeds based on morphologic and performance traits. Molecular studies have shown that nine of these breeds possess unique genetic lineages [44]. Outside China, yaks are typically named after the regions in which they are found [1].

In India, an estimated ≥70,000 yaks are concentrated in the states of Jammu and Kashmir, Himachal Pradesh, and Arunachal Pradesh. Ladakh, in Jammu and Kashmir, is home to the Arunachali breed, which is the only recognized breed in the country [45]. Pakistan’s Gilgit-Baltistan (GB) region supports an estimated 25,000 pure yaks and over 100,000 yak–cattle hybrids [3]. A recent census reported 14,914 yaks in northern Pakistan, with the highest concentration in Skardu. In contrast to the wild yak, which is a separate species but lacks detailed subgroup classification, the domestic yak exhibits multiple recognized subspecies [46]. The following is a list of wild and domestic yaks that are currently known (Table 1).

## 5. Genetic and Physiological Foundation of Production Traits of High-Altitude Adaptation in Yaks

Milk production is an important parameter influencing the development of the dairy industry and related economy [61]. Key economic traits include the somatic cell score (SCS), protein content, fat content, and overall milk yield. Conventional breeding techniques have improved many economically important traits, although, they have been less effective in improving milk yields due to their moderate heritability and polygenic traits [62]. In animal breeding, growth represents another crucial quantitative parameter that directly influences meat yields. Morphometric parameters, such as body height, length, chest circumference, and weight, are commonly measured to predict lifetime productivity and to inform breeding selection decisions [63]. Two processes that regulate development features depending on a variety of circumstances include changes in gene expression levels and genomic copy number variations (CNVs) [64]. Yaks have evolved adaptations to extreme climatic conditions, such as high altitudes, low temperatures, and intense UV radiation, which include the development of enhanced cardiopulmonary capacity, accelerated energy metabolism, and efficient oxygen transport mechanisms [31].

A candidate gene for the hypoxia-inducible transcription factor (HIF-2α) is endothelial PAS domain-containing protein 1 (*EPAS1*), which regulates the production of erythropoietin in response to the oxygen availability in the cellular microenvironment [65]. Identification and characterization of such genes will not only promote yak breeding programs but also offer insights into the genetic basis of hypoxia tolerance in mammals in high-altitude conditions. A comprehensive list of the genes linked to different production qualities in yaks is provided in Table 2.

## 6. Interspecies Hybridization Between Yaks and Cattle

Historical records document yaks interbreeding with domestic cattle, particularly in the regions of China and Central Asia. Native breeds of cattle, referred to as “yellow cattle” (*Bos taurus*) in China and *B. taurus* and *Bos indicus* (Zebu) in other places, were initially employed for interspecies hybridization. However, due to inherent reproductive barriers between species and low conception rates of the hybrids, crossbreeding between yaks and cattle was restricted to the F1 and F2 generations. Notably, hybrids in the F3 generation, containing approximately 12.5% yak DNA, generally lack the ability to thrive in the high-altitude conditions of the Qinghai–Tibet Plateau, which exceeds 3000 m [89,90]. Logistical constraints of artificial insemination (AI) and the high costs of maintaining genetically superior animals have limited the use of AI. However, AI has become the preferred method for mating yaks with improved breeds of cattle. This is due to behavioral incompatibilities, as yak cows do not allow bulls of other cattle species to approach them and vice versa under natural conditions. Hence, most crossbreeding programs rely on mating via AI [1].

Morphometric disparities between pure yaks and hybrid cattle evident in carcass weight are attributed to differences in live weight at the time of slaughter. Trial results have shown that F1 hybrids from improved breeds exhibit significantly accelerated growth during the first and second summers, gaining slaughter weight nearly 50% faster than pure yaks at 17 months of age [1]. Hybrids also yield superior meat and sufficient fat deposition, enhancing their commercial value. The milk yield among yak hybrids varies depending on the breed (crossbred or pure), location, and environmental factors. Generally, pure yaks produce the lowest volumes, native Pian Niu intermediate, and upgraded Pian Niu hybrids yield the highest. Normal hybridization practice involves crossing a yak female with a native cattle bull and using the resulting hybrid (Pian Niu) as a pack animal for draught purposes [90]. Reciprocal hybrids (false Pian Niu), produced by mating local female cattle with yak bulls, are mostly utilized for plowing. While female hybrids, both from F1 and backcross generations, exhibit normal fertility, male hybrids are typically sterile due to spermatogenic failure. Fertility may be restored only after multiple generations of backcrossing to either yaks or cattle lineages [1,90,91].

## 7. Microbial Threats to Yak Health

Bovine viral diarrhea virus (BVDV) is a significant cause of slow growth and mortality in yaks, resulting in considerable economic repercussions for yak husbandry [92]. Enterococci are facultative Gram-positive lactic acid-producing anaerobes found in the gut microbiota of various animals and are capable of withstanding extreme environmental conditions, such as pH, salt concentrations, temperatures, and antibiotics. Species such as *E. faecium* and *E. faecalis* have been isolated from yaks [93]. The presence of virulence factors and resistant genes in these bacteria poses a serious risk to yak health. Furthermore, the potential for biofilm formation poses additional challenges to veterinary treatment and zoonotic transmission, as within biofilms, antibiotic-resistant genes can be transferred horizontally, which are medicated by proteins such as Epa, Ebp, and PrgABC [93]. Therefore, systematic monitoring of biofilm formation potential, antimicrobial resistance (AMR) profile, and presence of virulence genes in enterococci isolated from yaks is important to safeguard animal and human health. Fatal hemorrhagic diarrhea, caused by pathogenic strains of *E. coli* in yaks, is a serious concern. Commensal *E. coli* strains in yaks can harbor diverse virulence genes and antibiotic resistance genes. These strains can cause asymptomatic infections and cross-contamination through water, food, carcasses, or feces, which poses a serious risk of zoonotic transmission [94].

## 8. Climate Change and Effects on Yak Productivity

Highland pastoral nomads rely heavily on yaks for subsistence and livelihood, since they are raised in high-altitude regions under transhumant systems across the Northeastern Himalaya. These habitats have an average temperature of 1.2 to 11.1 °C in winter and 7.9 to 19.7 °C in summer at a height of an average of 3000 m above sea level. During summer time, yaks commonly experience heat stress, particularly when the temperature–humidity index (THI) exceeds 52. To mitigate heat stress, especially when ambient temperatures rise above 13 °C, yaks elevate heart rates and respiration [95]. However, an estimated average increase in environmental temperatures of 0.01 to 0.04 °C per year at high altitudes poses a significant threat to yak health and productivity. Projections indicate that an increase of 2–3 °C could have catastrophic effects on high-altitude animals and ecosystems. Yaks who experience famine or loss in their physical condition due to the decrease in alpine meadows’ above-ground biomass and composition suffer infertility and decreased milk production. Additionally, global warming facilitates proliferation of pests and pathogens in lower permanent settlements in sub-alpine regions. Three potential strategies to mitigate heat stress in yaks are improved nutritional management, physical environmental changes, and the genetic development of strains that are less vulnerable to heat stress (Table 3).

Other climate change-related problems, such as the spread of vector-borne illnesses, are likely to make this situation worse. Vectors could then reside in larger regions due to changing climate conditions, and overcrowding brought on by a shortage of pasture may make animals more susceptible to disease transmission [95]. Quantifying changes in disease risk for yaks as a result of climate change is challenging due to the lack of baseline data on yak diseases [96]. The most obvious effect of climate change on yaks may appear to be the declining feed availability in traditional pastures, along with other socioeconomic variables [1]. It is necessary to conduct more research on how climate change affects yaks and concentrate on creating mitigation plans. These may include enhancing food and shelter provisions and promoting the use of yak–cattle hybrids. The traditional transhumant yak farming system is already impacted by climate changes. Rising THI values in summer and winter increase yaks’ thermoneutral threshold, adversely affecting the welfare, output, and performance of the animals. Furthermore, climate-driven changes are linked to an elevated risk of illness, which may intensify these adverse consequences on yak populations [96]. The expression and regulation of specific genes play crucial role in maintaining homeostasis under such adverse conditions (Table 4).

**Table 3:** Primers associated with different genes linked to different traits in yaks.

Country	Breeds and no of Individuals	Parameter	Gene	Associated Primers	Annealing Temperature	Base Pairs	Reference
China	➢ Pali/56; ➢ Gannan/187; ➢ Tianzhu white/288.	Hemoglobin concentration	*EPAS1*	EPAS1-S CGTGGTGACCCAAGATGGTG EPAS1-AGGTCACAGGGATGAGTGAAGTCAA GAPDH-S CCACGAGAAGTATAACAACACC GAPDH-A GTCATAAGTCCCTCCACGAT	60 60	573–691 422–542	[65]
China	➢ Cattle; ➢ Yaks; ➢ Cattle–yaks.	Spermatogenic arrest	*Dmrt7*	F: 5′-CCTCCAGATTGACTCTTAACTC-3′ R: 5′-GGACCCAAGGAAGGTAAGA-3′	64	1113	[71]
China	Cattle–yak/350	Milk fat	*SORBS1*	F: CACTTGCTCTCCCCTTCCTG R: CAACGTTCAGCCTCTGGACT F: ATGCCCTGTGCTGTCAACTT R: TACAGTGGTCGCTGCCATAC F: GGACAGGAGAGTTCTGTGGC R: AAGGACAGAGCTGCTGGAAC F: AGAGTGCCTCACTGCATGTC R: ACAGACTGGTGAACAGCCAC F: ACCGGATTGAGCCACAGTTT R: GGCACCAAGATTTTCCCAGC F: ACTGAGGTCTCTCAGCCAGT R: TACAGTGGTCGCTGCCATAC F: CTGTCTGACCCTGCTCTGTG R: GCCGGTGAGAAACTCAGGAA F: TGCCATCTCCTCCCTACACA R: GTCCACACCATGGCCACTAA F: CCAAGATGAGCACGGAAGGT R: GGGATTGTGGTGGTACCCAG F: TCTCCAGACATCCCGTGTGA R: GGTCTTGTGGGCATCCACTT F: GTTGAACGGATCTCCCCCAA R: GCAACTGGAAACTGCCCTTC F: AAGCCCCTAACCTTGGTGTG R: AGAGCACGTGCAGGCTAAAT	62 60 63 59 60 59 61 61 61 61 63 61	5791–6750 7741–8740 18,181–19,180 24,361–25,360 28,681–29,680 43,111–44,110 79,641–80,640 85,681–86,680 94,081–95,080 96,061–97,060 112,141–113,140 114,001–115,000	[70]
Pakistan	➢ Jiali ➢ Sibu ➢ Cawula/238	➢ Growth; ➢ Meat quality; ➢ Lactation.	➢ ACSL1_A2079T; ➢ ACSL1_G2409A; ➢ CAPN4_G-1222A; ➢ CYP4A11_G4806A; ➢ GHSR_T1387C; ➢ Hesx1_G618C; ➢ Hesx1_T226C; ➢ MyoD1_C1710T; ➢ OXGR1_A347G; ➢ TMEM-18_C1267T; ➢ TMEM-18_C4447T; ➢ UCP_ T1499C.	F: TTGATCAGGTGGCAGAGAAC R: CAAGGCTGATGACCATCAAC F: CGAGCTGTTCCAGTACTTTC R: TGCTTGGGATTGTGATCCTG F: GTCCCAAGACAAGTATCAGG R: TAAGACTGCGCATGTGCTTG F: GCTATGGACAGACATACTGG R: ACAAGTGATGGACTCTCCAG F: TTGAGCTACAACGTTGTCCC R: GTAGGGCATATGCTGTGTAG F: TAGAGGAGGACAGAATCCAG R: CTCAGATTAAACACAGAAAAC F: CTGTGTTCC ATC GACGAAAC R: CTCATGGGTGCACTTCATAC F: ACCCCTGCATACTAACCTAC R: TCAGAGCACCTGGTAAATCG F: ACATCTTCAAAATGCGGCCC R: ATGGCCCATCGCTTTTTGTG F: CTGTCTTCTCTCCCAGAA R: GGACACACAGCAGAAACAAG F: TGGACAAACAGCAGTGCAGG R: TCCTTCCTGAAAGCAACACC F: GGCAGAGTTCATGTATCTCG R: TTG AAGCCATGCACCTTGAG	52		[67]
Gansu, China	Gannan yak	1. Milk protein; 2. Milk fat; 3. Milk lactose; 4. Non-fat solid; 5. Total solid content.	*FASN* gene	F: CTGTCACCTTCCTCACTTGCCCT R: GAGGAGGAATCGGCCAGGATGTT F: CCCTCTAAAGCCGTCCTCACCA R: CCAGACCTTCATTTGCCAATCCTC F: ACAAGACAAGCCCGAGGAG R: TAGCAGGCAGTTCCGAGAG	72	390 220 203	[66]
China	*Bos grunniens*/81	Fat content	*DGAT1* K232A	F: 5′-GGCGGGGTGCGAACTAAG-3′ R: 5′-GCACAGCACTTTATTGACACATTC-3′.	55	1760	[68]
China	Yak (*Bos grunniens)* ➢ Yak male calves/6; ➢ Adult male yaks/10; ➢ Chinese Yellow cattle/8.	➢ Intramuscular fat (IMF) content; ➢ Tenderness; ➢ pH.	MSTN and CAST	MSTN: F: AAAGAGGGGCTGTGTAATGC R: ATGGTAATGACCGTTTCCGT CAST: F: CGTGCCTCGGACCTCTAT R: CGTCTTTATCCTTGGCTTCT	52~54	260 254	[72]
China	*Bos Grunniens*/387	Growth	*KLF6*	F: ATGCTCATGGGAAGGGTGTG R: CTTGGCACCAGTGTGCTTTC	55–60	82	[69]
China	*Bos grunniens*	*MyHC I* and *MyHC IIB* expression	ACTB	F: ATTGCCGATGGTGATGAC R: ACGGAGCGTGGCTACAG	60	177	[75]
			GAPDH	F: TCACCAGGGCTGCTTTTA R: CTGTGCCGTTGAACTTGC		126	
			UXT	F: AGGTGGATTTGGGCTGTAAC R: CTTGGTGAGGTTGTCGCTGA		170	
			TBP	F: GTCCAATGATGCCTTACGG R: TGCTGCTCCTCCAGAATAGA		82	
			YWHAZ	F: AATGTTGTAGGAGCCCGTAG R: CTGCTTGTGAAGCGTTGG		190	
			RPL13A	F: CAAGCGGATGAACACCAA R: GCAGCAGGAACCACCATT		192	
			SDHA	F: GGGAACATGGAGGAGGACA R: CCAAAGGCACGCTGGTAGA		188	
			RPS15	F: GACCTTCCGCAAGTTCACCT R: ACCACCTCGGGCTTCTCCAT		198	
			HPRT1	F: GTGATGAAGGAGATGGG R: ACAGGTCGGCAAAGAAC		79	
			PPIA	F: TTTTGAAGCATACAGGTCC R: CCACTCAGTCTTGGCAGT		98	
			HMBS	F: GAACAAAGGAGCCAAGAAC R: CAGAGGGCTGGGATGTAG		121	
			MRPL39	F: AAACCTTTGACCAAGTCCTGT R: TTCCTCTTTGAATGCCCTCTC		135	
			PPP1R11	F: CAGAAAAGACAGAAGGGTGC R: TTCCGAAGTTTGATGGTTAG		164	
			B2M	F: CTGAGGAATGGGGAGAAG R: TGGGACAGCAGGTAGAAA		80	
China	Datong yak/55	➢ Disease resistance; ➢ Production performance.	*TLR2*	F: GGACAATGCCACGTGCTT R: GCACTGATCTCAAGCTCCTCAAG F: TGAGGAGCTTGAGATCAGTG R: ACTGTGTATCCTTGTGCTGG F: CCTAGGTAATGTGGAGACG R: AAGGAGGCATCTGGTAGAG F: CCAGCACAAGGATACACAGT R: CTTCATGTACCACAGTCCGT F: TTCCTGTTGCTCCTGCTCAC R: GACCACCACCAGACCAAGAC	58 58 58 58 58	552 822 574 526 599	[77]
China	Ashidan yaks/335	➢ Withers height; ➢ Body weight; ➢ Chest girth; ➢ Body length.	*AHR*	F: TCATACCGGGCTCTTTGCAG R: GTACCCTGAACACCCGAAGG BTF3 F: AACCAGGAGAAACTCGCCAA R: TTCGGTGAAATGCCCTCTCG F: CACCCGTCTTCACCCATCAG R: TGCCTCCATGTGAACTTGCT F: CTTCCTGGGCATGGAATCCTG R: CAGCACCGTGTTGGCGTAG	58 58 54 54	223 166 164 103	[78]
China	Ashidan yaks/274	➢ Withers height; ➢ Body weight; ➢ Chest girth; ➢ Body length.	*HSF1*	F: TCCGGAGGTGGTCCACAT R: GAACTCGGTGTCATCCCTCTCT F: CCATCATCTCCGACATCACC R: CTCCTCCTTTACGCGAACC F: ATTGCCGATGGTGATGAC R: ACGGAGCGTGGCTACAG	58 63.3 55	290 113 177	[80]
China	➢ Datong yaks/222; ➢ Polled yaks/165; ➢ Tianzhu yaks/30; ➢ Gannan yaks/30; ➢ Plateau yaks/30.	➢ Body height; ➢ Body length; ➢ Chest girth; ➢ Body weight; ➢ Cannon width.	*CHKB*	F: GCAGTCTCGGTTCCAGTTCT R: AATGCAAGGAGTCGGAGGTG F: AACCAGGAGAAACTCGCCAA R: TTCGGTGAAATGCCCTCTCG F: AGCTAATCGGTATGCCCTGG R: AACTGGAACCGAGACTGCG F: ATGAAAGGGCCATCACCATC R: GTGGTTCACGCCCATCACA	60.57 59.32 55.40 57.45 60.18 60.11 55.85 60.00	90 166 118 204	[81]
China	Ashidan yaks/336	➢ Withers height; ➢ Body weight; ➢ Chest girth; ➢ Body length.	*HPGDS*	F: ATCCGGGCACTGTTAGAAGG R: GCCTGCAAAGTCTGTACTGT F: AACCAGGAGAAACTCGCCAA R: TTCGGTGAAATGCCCTCTCG F: ACCTGCCCATTTCTATCCTGAC R: ACTGTTTCTTAGCCCATCGCAT F: AATGAAAGGGCCATCACCATC R: GTGGTTCACGCCCATCACA		170 166 187 204	[79]
China	Ashidan yaks/350	➢ Withers height; ➢ Body weight; ➢ Chest girth; ➢ Body length.	*CADM2*	F: GACTTCCCAGGATTGCCTGT R: CCCTGGGAGCACAGTTGTTT F: GGCTGTCACGTTCTTCTCTCA R: AGGGTTCATCCTGGAGGCTT F: AACCAGGAGAAACTCGCCAA R: TTCGGTGAAATGCCCTCTCG	62	186 196 166	[82]
China	Ashidan yaks/311	Body weight; Withers height; Body length; Chest girth.	*SOX6*	F: GCAACTACCACACCGTCACCTC R: TCCGCCGTCTGTCTTCATACCA F: AACCAGGAGAAACTCGCCAA R: TTCGGTGAAATGCCCTCTCG F: CGTTTGGGCAGGAGTTTGGA R: CGTTTGGTGGCTGTGGAGTT F: GCAGGTCATCACCATCGG R: CCGTGTTGGCGTAGAGGT	59 59 60 60	114 166 148 158	[97]
China	Ashidan yaks/315	Body weight; Withers height; Body length; Chest girth.	*MICALL2* *MOGAT2*	F: CCGTCGTCTAATGCCAGTGA R: CATCTTTCCGCTGGACGGTA F: CGCTGGTCAAGACTGCCTAT R: ACAGTGAGGAAAACCCGGTG F: AACCAGGAGAAACTCGCCAA R: TTCGGTGAAATGCCCTCTCG F: CCTCATGGTGGACTGGTTCC R: CAATGATGTCGCTTCGGCTG F: CGCTGGTCAAGACTGCCTAT R: CATCATCAGATGTGGGCGGA F: AATGAAAGGGCCATCACCATC R: GTGGTTCACGCCCATCACA	58.0 60.0 60.0 59.9 59.8 58.8	133 126 166 239 155 204	[98]
China	Ashidan yaks/326	Body weight; Withers height; Body length; Chest girth.	➢ *SOX5* ➢ *SOX8*	F: AACCAGGAGAAACTCGCCAA R: TTCGGTGAAATGCCCTCTCG F: GCTTCCCAGTTCGCTTAG R: TTTCTGCCTTGGATGCTC F: CCTTGGGTCACTCGGGTTG R: GCGGCTCGGATTCTTTCG F: CCACGAGAAGTATAACAACACC R: GTCATAAGTCCCTCCACGAT F: AAGAAACTGGCTGCGTCTCA R: TAATGGCGGCAGTTGACCTT F: CCGCACATCAAGACGGAGCA R: TGACGGGTAGCCAGGGAACG	63 55.6 63 56.1 56.1 64	166 104 141 120 168 213	[85]
China	Yaks/354	Body weight; Average daily gain.	*MC4R*	F: 5′-TGGGA CATTTATTCACAGCAG-3′ R: 5′CCTACACAG AAGAAAAAGCT-3′	55	1238	[86]

**Table 4:** Genes of yaks associated with stress to environmental factors.

Genes	Functions of Genes Associated with Environmental Stress	References
*MMP3*	As a master regulator of the cellular response to hypoxia, hypoxia-inducible factor-1α is thought to have matrix metalloproteinases-3 (MMP3) as one of its primary target genes.	[99,100]
*ATP8*; *ATP6*.	➢ An inner membrane polypeptide of the F_0_ component, subunit 8 of the mitochondrial F_1_F_0_-ATP synthase (ATP8) is necessary for the correct assembly of the ATP synthase holoenzyme. ➢ The mitochondrial genomes of every eukaryotic organism that has been investigated to date encode subunit 6 of the mitochondrial F_1_F_0_-ATP synthase (ATP6), which is an inner membrane polypeptide similar to ATP8. One essential part of the proton channel is ATP6.	[101]
*HIF-1*	Because it regulates the localized tissue hypoxia that takes place in these settings, hypoxia-inducible factor 1 (HIF-1) has been found to have a significant role in the pathophysiology of tumor vascularization, myocardial ischemia, and stroke.	[102]
*AQP4*	The production of brain edema is one of the several physiopathological processes in which aquaporin-4 (AQP4) is implicated. Additionally, it controls calcium signaling, waste removal, potassium buffering, and extracellular space volume.	[103]
*EPAS1*	One essential transcription factor that controls the expression of genes involved in oxygen sensing is the endothelial PAS domain protein 1 gene (EPAS1).	[65]
*VEGF-A*	The vascular endothelial growth factor-A gene (VEGF-A), a crucial regulator of angiogenesis and endothelial cell mitogen, plays a major role in adaptation to high altitudes.	[104]
*HIF-1α*	*HIF-1α* is the oxygen-regulated subunit of HIF-1 that controls the transcription of genes related to oxygen homeostasis in response to hypoxia.	[105]
*LDH*	An essential component of anaerobic metabolism, lactate dehydrogenase (LDH) catalyzes the transformation of pyruvate to lactate during glycolysis in mammals.	[106]
*HIFs*	Oxygen-dependent transcriptional activators known as hypoxia-inducible factors (HIFs) are critical for mammalian development and tumor angiogenesis. In reaction to hypoxia, they control the transcription of genes related to oxygen homeostasis.	[107]
➢ *ALDH4A1*; ➢ *ALDH2*; ➢ *ECI1*.	The primary roles of ALDH4A1, ALDH2, and ECI1 in bioenergy metabolism under hypoxic settings suggest that they might be engaged in hypoxia adaptation processes.	[108]
➢ *COL1A2*; ➢ *COL3A1*; ➢ *COL5A2*; ➢ *COL14A*; ➢ *COL15A1*.	The crucial involvement of collagen-related pathways in high-altitude adaptation is highlighted by five collagen genes: COL1A2, COL3A1, COL5A2, COL14A1, and COL15A1.	[109]
➢ *COX5A*; ➢ *UQCRC1*; ➢ *CAP*; ➢ *CHRM2*.	In the heart-related modules of yaks, UQCRC1 and COX5A are frequently found to be differentially expressed hub genes linked to the energy source for cardiac contraction. The lung-related module also contains the common differential hub gene CAPS, which is connected to the contraction of the smooth muscle in the pulmonary arteries. Furthermore, the heart of yaks contains a unique hub gene called CHRM2, which is differentially expressed and essential for the independent control of cardiac function.	[110]
➢ *MAPKAPK3*; ➢ *PXN*; ➢ *NFATC2*; ➢ *ATP7A*; ➢ *DIAPH1*; ➢ *F2R*.	It is suggested that MAPKAPK3, ATP7A, PXN, NFATC2, DIAPH1, and F2R are new and intriguing options for controlling hypoxia adaptation in the heart.	[111]
➢ *MT-ND1*; ➢ *MT-ND2*.	Two mitochondrial genes, MT-ND1 and MT-ND2, encode subunits of NADH dehydrogenase, which are associated with high-altitude adaptation and are necessary for the electron transport chain in oxidative phosphorylation (OXPHOS).	[112]
*CSF_2_*	By regulating the production of the heat shock protein 70 kDa 1A, colony-stimulating factor 2 (CSF2) is known to support the growth and survival of preimplantation embryos in rats and ruminants.	[113]

## 9. Conclusions

Yaks (*Bos grunniens*) are genetically distinct and ecologically very important species that are uniquely adapted to the high-altitude rangelands of Asia. Their socioeconomical role underscores the urgency of safeguarding these populations in response to climate changes, declining forage, and emerging diseases. Selective breeding and better management practices by integrating genetics with ecological and health approaches will sustain these yak populations and dependent human communities. Furthermore, this review has also provided a summary of various breeds, both naturally occurring and produced through hybridization.

## Figures and Tables

**Table 1:** Description of the available yak breeds.

Sr No.	Breed Name	Country	Location	Characteristics	Reference
1.	Afghanistan yak	Afghanistan	West Asia	▪ They flourish at high elevations with low air pressure and oxygen levels. ▪ Additionally, they can graze on sheep-grazed short grasslands.	[47]
2.	Merakpa yak	Bhutan	Eastern Bhutan, Tibet	▪ Body is smaller and lighter. ▪ Smaller in size and colored black and white or brown.	[48]
3.	Haapa yak		Western and central Bhutan, Tibet	▪ Haapa is used as a draught and transport animal and for meat, milk, and hair. ▪ The hairs are used to make garments, ropes, and tents. ▪ Lastly, details regarding Yak genes involved in various production qualities, such meat and milk, and how these genes have adapted to the high altitudes are included in the review. They are usually black, but less than 5% are white or albino. Other prevalent hues are black and white or brown. Most have black coats and are horned.	
4.	Datong yak	China	East Asia	▪ Generally black, although there may be a few brown hairs present. ▪ Daily milk yield is 1.40 kg. ▪ Males are horned, while females can be either horned or hornless.	[1]
5.	Huanhu yak		Qinghai Lake	▪ Lastly, details regarding yak genes involved in various production qualities, such meat and milk, and how these genes have adapted to the high altitudes are included in the review. ▪ They are usually black, but less than 5% are white or albino. Other prevalent hues are black and white or brown. Most have black coats and are horned.	[1]
6.	Guoluo yak		East Asia	▪ Used for milking. ▪ Daily milk yield is 1.06 kg.	[49]
7.	Batang yak		Batang area, Qinghai Province	▪ Used for their milk. ▪ Daily milk yield is 3.18 kg.	
8.	Gannan yak		Gannan Tibetan, Gansu	▪ Strong muscular body and black in color. ▪ Strong body structure with well-developed muscles. Black is the predominant color, although some individuals may have white spots on black, gray, or yellow and white. ▪ Daily milk yield is 1.78 kg.	
9.	Heihe yak		East Asia	▪ Used for milking. ▪ Daily milk yield is 2.67 kg.	
10.	Bazhou yak			▪ The Bazhou yak is characterized by excellent meat production traits and a strong ability to adapt to environmental conditions. ▪ Body is large and rectangular. ▪ Black is the primary color, although some are black and white, brown, or gray and white. ▪ The majority have fine, long horns. ▪ Daily milk yield is 2.56 kg.	[50]
11.	Jiulong yak		Sichuan Province, Jiulong	▪ Their bodies are huge and tall, and they are typically black or black and white. ▪ Most of them have long, beautiful horns. ▪ The production of milk each day is 2.31 kg.	[51]
12.	Maiwa yak		East Asia	▪ Most of the animals have horns, which direct rearward and bend inward. ▪ Daily milk yield is about 2.43 kg. ▪ The remaining ones are medium-sized and black or black and white, brown, cyan (a very dark blue), and black with white spots on the head and tail.	
13.	Niangya yak, Liangya			▪ The maximum milk yield occurs between June and August, when grass grows abundantly; however, the milk–fat ratio is low during this period. ▪ Most are pure black. ▪ Used for milking.	
14.	Jinchuan yak		Maori and Akeli Village, Sichuan Province, Jinchuan	▪ They produce high-quality milk and a significant yield of meat. ▪ They are robust and possess greater resilience to natural pressures.	[52]
15.	Sarlag yak		East Asia	▪ They flourish at high altitudes, where atmospheric pressure is low and oxygen levels in the air are reduced.	[48]
16.	Muli yak			▪ A large body, either entirely black or black with white spots. ▪ Both sexes are horned. ▪ Daily milk yield is 1.06 kg.	[47]
17.	Sibu yak, Tibetan high-mountain yak			▪ Large, rectangular body and horns. ▪ Mountain and grassland yaks are classified into two types based on body conformation and appearance, characterized by horn shapes that either “hold the head” or spread outwards and upwards, respectively. ▪ Daily milk yield is 1.0–1.5 kg.	
18.	Kyrgyz yak			▪ They can also graze on short-grass areas grazed by sheep. ▪ Known for high-quality meat. They are highly adaptable to high altitudes, low atmospheric pressures, and air with a low oxygen content.	
19.	Plateau yak of Qinghai			▪ The genes of wild yaks have been incorporated into this breed, resulting in some characteristics similar to those of wild yaks, including enhanced meat and milk production.	
20.	Xingjiang yak			▪ Well-suited for alpine regions at altitudes between 2400 and 4000 m. ▪ Their entire body is covered in long hair, with the belly featuring skirt-shaped hair and broom-shaped hair on the tail. They are primarily black, brown, or gray in color. ▪ Daily milk yield is 2.6 kg.	
21.	Shandang yak			▪ Known for its milk.	
22.	Tianzhu white yak			▪ Pure white coat.	
23.	Zhongdian yak			▪ Strong and muscular build with a wedge-shaped body type. ▪ Most of the animals are black (62.4%), with 27.5% displaying a black-and-white coloration, while the remainder have black fur with white spots on their forehead, legs, and tail. ▪ Both sexes possess horns, which are long and slender, either black or gray in color, extending outwards and upwards. The tips of the horns are oriented either forward or backward. ▪ Daily milk yield is 1.68 kg.	
24.	Jiali/Alpine yak			▪ Spotted, pure black, white, brown, or gray are some of the different colors. ▪ Body is large. ▪ Mostly horned (83%), with a wide distance between the bases. ▪ Daily milk yield is 0.82 kg.	
25.	Pali yak		Pali town, Yadong, Rigeze	▪ Rectangular, strong body. ▪ Mostly all black; however, deep brown, yellow–brown, and black and white may also be found. ▪ Daily milk yield is 1.6 kg.	[1]
26.	Arunachali yak	India	Northeastern states, India	▪ Medium-sized and mostly black, Arunachali yaks are distinguished by their long, thick hair that falls down their bodies and their kind disposition. They possess a convex head, horizontal ears, and prominently curved horns with pointed tips. ▪ Males have larger horns than females, and they are mainly curled and black in hue. ▪ Milk yield per day is 1 kg.	[53]
27.	Chour-gau yak		Ladakh	▪ They flourish in environments with high heights, low atmospheric pressure, and low oxygen levels. ▪ Additionally, they can graze on sheep-grazed short grasslands.	[48]
28.	Indian yak		South Asian	▪ Four breeds: Ladakhi or Changthang, Himachali, Garhwali, Arunachali. ▪ Coat colors and patterns vary. ▪ Wide variability in phenotypes and color patterns.	[47]
29.	Altai yak	Mongolia	East Asia	▪ Most have well-developed horns. Males can have horns of 50 to 100 cm in length. ▪ The body is of an alpine type, and their long body is covered with thick hairs. ▪ Body coat is mainly black or black and white.	[54]
30.	Hangai yak			▪ Inhabitants of Hangai mountains and woodland pastures at elevations of 1800–3000 m. ▪ Large-framed with varied coat colors. ▪ Color of body coat varies greatly. ▪ Used for transport, meat, and milk.	
31.	Khainag yak			▪ This breed features long legs with broad strides, making them easy to train for transportation. They are calm, persistent, and capable of leading the herd across water and snowdrifts. ▪ Milk yield per lactation is 470 kg.	[55]
32.	Nepalese ak	Nepal	South Asia	▪ They are raised in 3000 m above sea level in trans-Himalyan region.	[56]
33.	Siru yak	Pakistan		▪ This breed is smaller in size than the Chanthangi yak, with a slenderer build and shorter hair. ▪ Siru yaks are primarily utilized for transportation, serving as pack animals and providing valuable resources, such as wool, milk, and meat.	[57]
34.	Nagor yak			▪ It is known for its adaptation to high altitudes and harsh climates.	[44]
35.	Balti yak			▪ They are known for their meat and wool production and are commonly used for transportation in the region.	[56]
36.	Himalayan yak			▪ This breed of yak has a thick coat of hair that ranges in color from black to brown. ▪ It is well-adapted to the harsh weather conditions of the region.	[58]
37.	Nubra yak			▪ This breed of yak is known for its long and soft hair, which is used to make shawls and other clothing items. ▪ The Nubra yak is also used as a pack animal in the region.	[3]
38.	Mishmi yak			▪ They are well-adapted to the rugged terrain and cold climate and are used for milk, meat, and transportation.	[10]
39.	Gaddi yak			▪ They are primarily used for transportation but also for milk and meat.	
40.	Baltistani yak			▪ It is known for its hardiness and ability to thrive in high-altitude environments.	[3]
41.	Kharmangi yak			▪ It is known for its excellent milk production and is often used for dairy purposes.	[58]
42.	Gilgit yak			▪ It is known for its hardiness and is often used for transportation purposes.	[59]
43.	Skardu yak			▪ It is known for its large size and strength, which make it a good draft animal.	
44.	Pakistani yak			▪ Inhabit altitudes higher than 3000–7000 m above sea level.	[48]
45.	Russian Federation yak	Russia	Northern Asia	▪ They thrive at high altitudes, in low atmospheric pressures, and in air with a low oxygen content.	[47]
46.	Tajikistan yak	Tajikistan	Altai Territory, Tyan Shan	▪ They flourish in high-altitude environments characterized by low atmospheric pressure and reduced oxygen levels in the air. ▪ They can also feed on short-grass areas that are grazed by sheep. ▪ Yaks are highly efficient at utilizing natural steeper terrains that are unsuitable for other animals.	[5]
47.	Tongde yak	Tongde County of Qinghai Province	East Asia	▪ Possessing strong adaptations and resistance characteristics for high-altitude environments, the population size is approximately 250,000 yaks.	[60]

**Table 2:** Genes or SNPs associated with different traits in yaks.

Location	Species	Trait Studied	Gene/SNP	Function of Associated Gene	Tissues	Method	Biological Impact	Origin	Ref.
Gannan–Tibetan Autonomous Prefecture, Gansu	Gannan yak/290	Total solid content, Milk fat, Milk protein, Non-fat solids, and Milk lactose.	*FASN*	An enzyme called fatty acid synthase aids in the production of fatty acids (FAs) and is essential for mammalian de novo lipogenesis.	➢ Kidney; ➢ Rumen; ➢ Lung; ➢ Spleen; ➢ Large intestine; ➢ Liver; ➢ Jejunum; ➢ Heart; ➢ Subcutaneous fat; ➢ Mammary gland; ➢ Abomasum.	PCR-SSCP	The mammary gland and subcutaneous fat had the highest expression levels of fatty acid synthase, whereas the heart, small intestine, lung, kidney, abomasum, rumen, large intestine, longissimus dorsi muscle, and liver had the lowest expression levels.	China	[66]
Nagqu Jiali County, Tibet; Muzugongga County, Tibet; Neirong County, Tibet	Jiali Sibu Cawula/238	Growth meat quality and lactation	ACSL1_A2079T ACSL1_G2409A CAPN4_G-1222A CYP4A11_G4806A GHSR_T1387C Hesx1_G618C Hesx1_T226C MyoD1_C1710T OXGR1_A347G TMEM-18_C1267T TMEM-18_C4447T UCP_ T1499C		Venous blood	Snapshot technology	The genetic diversity of the three yak populations was well preserved, and none of the populations had undergone artificial selection for economic traits.	Pakistan	[67]
Sichuan Province	Jiulong yak/32	Milk fat content	*DGAT1 K232A* polymorphism	A key player in cellular triglyceride metabolism, diacylglycerol O-acyltransferase 1 (DGAT 1; EC 2.3.1.20) catalyzes the last stage of triglyceride production. It has a role in lactation, adipose tissue development, and intestinal fat absorption. Furthermore, it has the ability to catalyze the creation of diacylglycerols, waxes, and retinyl esters in vitro.	Longissimus muscle	Identification of DGAT1 gene splicing and assay of isoform proportion	There was no discernible difference between the liver and biceps femoris in yaks and cattle.	China	[68]
						Analysis of DGAT1 mRNA levels was conducted using quantitative real-time RT-PCR.	The liver and adipose tissue of adult yaks had noticeably greater levels of DGAT1 mRNA than skeletal muscles, such the biceps femoris and longissimus dorsi.		
	Jiulong yaks/58 Zhongdian yak/23				➢ Longissimus muscle ➢ Whole milk.	PCR-SSCP;	PCR-SSCP analysis and direct sequencing of the PCR products revealed three genotypes of DGAT1: AA, GC, and AA/GC. Among the 81 samples, only one yak displayed the AA/GC genotype, while the others exhibited the AA genotype.		
Datong Yak Farm, Qinghai Province Gansu Province, Tianzhu Tibetan Autonomous County Qinghai Province Datong Yak Farm, Qinghai Province Gansu Province, bordering Sichuan and Qinghai	*Bos Grunniens*/387 Polled Tianzhu white plateau Datong Gannan	Growth	*KLF6*	A zinc finger transcription factor that is expressed in a variety of tissues, KLF6 belongs to the Kruppel-like factor family. It is essential for cell division, proliferation, development, and growth-related signaling pathways.	➢ Heart; ➢ Liver; ➢ Kidney; ➢ Spleen; ➢ Skeletal; ➢ Lung; ➢ Brain; ➢ Muscle; ➢ Adipose fat.	RT-qPCR	It is evident that KLF6 CNVRs play a major role in regulating the gene’s mRNA expression levels in the skeletal muscles of *Bos grunniens.* Additionally, there is a negative association between DNA copy numbers and gene expression, indicating that the expression of this gene influences quantitative growth features in yak populations.	China	[69]
Ngawa Tibetan and Qiang Autonomous Prefecture, Hongyuan County, Sichuan Province	Cattle yak/350	Milk fat	*SORBS1*	The Cbl-associated protein encoded by the sorbin and SH3 domain-containing 1 (SORBS1) gene is crucial for insulin signaling and stimulation. It is a member of the SORBS family.	➢ Blood; ➢ Milk.	PCR	The SORBS1 gene is a possible genetic marker for selecting milk fat qualities in cattle and yaks, since polymorphisms in this gene are highly correlated with these features. All nine of the SNPs that were found showed a strong association with the cattle yak’s milk fat traits.	China	[70]
Nil	Pali/56 Gannan/187 Tianzhu White/288	Hemoglobin concentration	*EPAS1*	The endothelial PAS domain protein 1 gene (*EPAS1*) is a key transcription factor that controls the expression of genes associated with oxygen levels.	➢ Heart; ➢ Kidney; ➢ Muscles; ➢ Ovary; ➢ Pancreas; ➢ Liver; ➢ Lung; ➢ Spleen;	RT-PCR	The lungs, kidneys, liver, heart, ovaries, spleen, muscles, and pancreas contain the highest quantities of EPAS1 mRNA in yaks.	China	[65]
Gannan Autonomous Prefecture, Gansu Province	Cattle Yaks Cattle-yaks	Spermatogenic arrest	*Dmrt7*	Dmrt7 appears to be exclusive to mammals and is expressed only in the adult testes and embryonic gonads. It has no bearing on female gametogenesis but is essential for male gametogenesis.	➢ Testis; ➢ Lung; ➢ Kidney; ➢ Muscle; ➢ Liver; ➢ Spleen; ➢ Ovary; ➢ Epididymis; ➢ Heart.	RT-PCR	Although there was no discernible difference in the amounts of the Dmrt7 protein between the testes of cattle and yak, the expression of the Dmrt7 protein in cattle–yak was much lower than that in cattle and yak. In cattle and yak, male sterility is associated with this decreased expression of Dmrt7.	China	[71]
Nil	*Bos grunniens* Yak male calves/6 Adult male yaks/10 Chinese yellow cattle/8	➢ Intramuscular fat (IMF) content; ➢ Tenderness; ➢ pH.	MSTN ➢ CAST	As negative regulators of skeletal muscle development, myostatin (MSTN) and calpastatin (CAST) are potential genes associated with muscle growth and tenderness.	Longissimus muscles	RT-PCR	Despite being smaller in body size than yellow cattle, adult yaks exhibited lower levels of MSTN and similar levels of CAST mRNA in the longissimus muscle compared to yellow cattle.	China	[72]
➢ Tibet, Lhari County; ➢ Tibet, Yadong County; ➢ Tibet, Maizhokunggar County.	*Bos grunniens*/480 Pali yak (YD) Sibu yak (SB) Jiali yak (JL)	Body weight Parathyroid hormone (PTH) Adrenomedullin	G protein-coupled receptor kinase 4 (GRK4) males: ➢ AX-174402854; ➢ AX-174929694; ➢ AX-174547362; ➢ AX-174734142; ➢ AX-174706158; ➢ AX-174783962; ➢ AX-174627015; ➢ AX-174702570; ➢ AX-174961896; ➢ AX-174407967; ➢ AX-174928167; ➢ AX-174555047. ➢ Females: ➢ AX-174845027; ➢ AX-174891371; ➢ AX-174570649; ➢ AX-174620133.	Numerous studies have linked muscular dystrophy and obesity to G protein-coupled receptor kinase 4 (GRK4).	Venous blood	Enzyme linked immunosorbent assay (ELISA)	Yaks’ PTH and ADM levels were measured, and the results show that PTH levels and body weight were positively correlated, whereas ADM levels and body weight were negatively correlated. Additionally, there were differences in the AX-174555047 mutation. By modifying GRK4 expression, the SNP AX-174555047 may have an impact on body weight, which in turn impacts PTH and ADM function.	China	[73,74]
Qinghai Datong Yak Farm	*Bos grunniens*	➢ *MyHC I*; ➢ *MyHC IIB* expression.;	*MyHC*	About 35% of muscles’ protein content is made up of myosin heavy chain (MyHC), the main structural protein. The skeletal muscle of several mammalian species contains four adult MyHC isoforms: MyHC I, IIA, IIX, and IIB.	Skeletal muscles: ➢ Trapezius pars thoracica; ➢ Extensor digitorum lateralis; ➢ Gluteobiceps; ➢ Gastro cnemius; ➢ Fibularis longus; ➢ Semitendinosus; ➢ Psoas major; ➢ Latissimus dorsi; ➢ Supraspinatus; ➢ Longissimus dorsi muscle.	RT-qPCR	While GAPDH, the most often used reference gene, displayed the greatest fluctuation in expression across various muscle tissues, UXT and PRL13A were shown to be the most stable reference genes. The muscles with the highest concentration of type I muscle fibers and the lowest concentration of type IIB muscle fibers were the psoas major (Chapman), trapezius pars thoracica (TPT), and extensor digitorum lateralis (EDL). Conversely, the largest percentage of type IIB muscular fibers was found in the gluteobiceps (GB) muscle.	China	[75]
Longri Breeding Farm of Sichuan Province	Maiwa yaks/406	Body weight	➢ *MFSD4*; ➢ *LRRC37B*; ➢ *NCAM2*.	*MFSD4* has consistently shown a significant impact on the main intake effects in skeletal muscle. *LRRC37B* has been reliably associated with body size in pigs, while neural cell adhesion molecule 2 *(NCAM2)* has been demonstrated to correlate with body weight in Simmental cattle.	Blood	GWAS	Seven markers were found to be significantly associated with the body weight trait. Among these, several candidate genes, including MFSD4, LRRC37B, and NCAM2, were identified.	China	[76]
Datong Yak Breeding Farm in Qinghai Province	Datong yak/55	➢ Disease resistance; ➢ Production performance.	*TLR2*	Toll-like receptors (*TLR*s) are important pattern recognition receptors and are widely expressed on the surfaces of innate immune system cells, such as monocytes and macrophages.	Whole blood jugular vein	PCR	The protein plays an important role in the body’s immune regulation mechanism.	China	[77]
Datong Yak Farm, Qinghai Province	Ashidan yaks/335	➢ Body length; ➢ Body weight; ➢ Withers height; ➢ Chest girth.	*AHR*	The basic helix–loop–helix PAS family includes the ligand-dependent transcription factor known as the aromatic hydrocarbon receptor (AHR). It serves as an environmental sensor that is conserved throughout a variety of biological evolutionary processes.	➢ Liver; ➢ Spleen; ➢ Lung; ➢ Kidney; ➢ Adipose tissue; ➢ Blood; ➢ Heart; ➢ Muscle.	qPCR	The liver, heart, adipose tissue, kidneys, spleen, and lungs had the highest levels of AHR expression.	China	[78]
Datong County, Qinghai Province	Ashidan yaks/336	➢ Chest girth; ➢ Body weight; ➢ Body length; ➢ Withers height.	*HPGDS*	In male reproduction, HPGDS contributes to the negative control of cell proliferation through its involvement in PGD2 formation. As a potential gene, the HPGDS gene is also linked to characteristics of chicken meat quality.	➢ Muscle; ➢ Adipose tissue; ➢ Blood; ➢ Lung; ➢ Kidney; ➢ Heart; ➢ Liver; ➢ Spleen.	PCR	In general, the 30-month-old yak had a higher level of HPGDS gene expression than the 6-month-old yak.	China	[79]
Datong Yak Farm, Qinghai Province	Ashidan yaks/274	➢ Body weight; ➢ Withers height; ➢ Body length; ➢ Chest girth.	*HSF1*	*HSF1* is expressed in the cardiomyocytes, tissues, and organs. It exerts an irreplaceable effect in anti-apoptosis, anti-inflammatory, and anti-ischemia-reperfusion injury of cardiomyocytes. Furthermore, HSF1 is significant for the normal development of the body.	➢ Kidney; ➢ Blood; ➢ Lung; ➢ Muscle; ➢ Heart; ➢ Liver; ➢ Spleen; ➢ Adipose tissue.	qPCR	HSF1 relative expression in muscles, followed by heart, liver, kidney, adipose tissue, lung, and spleen	China	[80]
Datong Yak Farm in Qinghai Province; ➢ Datong Yak Farm in Qinghai Province; ➢ Tianzhu Tibetan; Autonomous County in Gansu Province; ➢ Gansu Province, bordering Sichuan and Qinghai ➢ Northern and southern Qinghai Province.	Datong yaks/222 Polled yaks/165 Tianzhu yaks/30 ; Gannan yaks/30	➢ Cannon width; ➢ Body height; ➢ Chest girth; ➢ Body weight; ➢ Body length.	*CHKB*	The CHKB gene is essential for maintaining normal mitochondrial function and plays a key role in the biosynthesis of phosphatidylcholine. It also regulates osteoclast and osteoblast functions, contributes to meat production and quality, supports growth and muscle development, and maintains bone homeostasis. Additionally, CHKB is involved in eye movement and the regulation of wakefulness.	➢ Heart; ➢ Liver; ➢ Lung; ➢ Blood; ➢ Skeletal muscle; ➢ Adipose tissues; ➢ Brain; ➢ Spleen; ➢ Kidney.	qPCR	In 90-day-old fetuses, the CHKB gene was highly expressed in the lungs, brain, spleen, and kidneys; moderately expressed in the liver and muscle tissues; and showed low expression levels in the heart. In contrast, at the adult stage, CHKB expression was significantly higher in adipose, spleen, and lung tissues compared to other tissues. Moderate expression was observed in muscle and brain tissues, while the remaining tissues exhibited only low expression levels.	China	[81]
Datong Yak Farm, Qinghai Province	Ashidan yaks/350	➢ Body weight; ➢ Withers height; ➢ Chest girth; ➢ Body length.	*CADM2*	Variants of the CADM2 gene have been previously recognized as playing a vital role in influencing human body mass index (BMI) values via the central nervous system. Additionally, analyses in mice have revealed that CADM2 is closely associated with body weight and energy homeostasis through brain activity.	Blood	qPCR	The CNV2 mutation significantly influenced body weight in yaks at six months of age.	China	[82]
Tianzhu white yak propagation bases of Wuwei City, Gansu Province	Yak	Hair follicles’ (HFs) cycle	➢ Differently expressed long noncoding RNA (DELs); ➢ Differently expressed mRNA (DEMs).	-------------------------	➢ Small intestine; ➢ Heart; ➢ Skin; ➢ Liver; ➢ Kidney; ➢ Subcutaneous fat; Muscle; ➢ Spleen; ➢ Lung; ➢ Testis.	RT-qPCR	Hub genes, including FER, *ELMO1*, *PCOLCE*, and *HOXC13*, were identified through screening in various modules.	China	[83]
Qinghai Province, Datong Yak Farm in Qinghai	*Bos grunniens*/536	Growth traits Gene expression	*GPC1*	The *GPC1* gene plays a crucial role among proteoglycans in differentially regulating muscle cell proliferation, differentiation, and cellular responsiveness to FGF2. Notably, the copy number variations (CNVs) of the *GPC1* gene are associated with meat production and quality, which are economically important traits that have been thoroughly considered for artificial selection in yak breeding.	➢ Blood; ➢ Skeletal muscle; Heart; ➢ Liver; ➢ Lung; ➢ Brain; ➢ Spleen; ➢ Kidney; ➢ Adipose fat.	qPCR	*GPC1* exhibited significantly high expression levels in muscle and spleen tissues; moderate expression in the brain and lungs; and weak expression in the liver, kidneys, and heart.	China	[84]
Datong Farm, Qinghai Province	Ashidan yaks/326	➢ Body weight; ➢ Withers height; ➢ Body length; ➢ Chest girth.	➢ *SOX5* *SOX8*	Normal development and bone formation depend on the SOX5 and SOX8 genes.	➢ Heart; ➢ Spleen; ➢ Liver; ➢ Lung; ➢ Kidney; ➢ Muscle.	qPCR	Compared to the heart, spleen, kidney, and muscles, the expression of SOX5 was substantially higher in the lung. In a similar vein, SOX8 expression in the lung was noticeably greater than that in the muscles and liver.	China	[85]
Maiwa yak in Hongyuan County, Sichuan Province	Yaks/354	➢ Body weight; ➢ Average daily gain;	➢ *MC4R*	*MC4R* (melanocortin 4 receptor) is expressed in the appetite-regulating areas of the brain and is involved in leptin signaling pathways.	➢ Ear muscle	PCR	*SNP4* was associated with significant changes in the seventh transmembrane domain of the *MC4R* protein, leading to functional deterioration or even loss of function of *MC4R*. This may contribute to increased feed intake, body weight, and average daily gain in yaks with CC genotypes.	China	[86]
Qilian County, Qinghai Province	*Bos grunniens*/423	➢ Body weight; ➢ Body length; ➢ Withers height; ➢ Chest circumference.	➢ *GH1*	It regulates essential cellular and physiological processes by binding to various hormones of the somatotropic axis, influencing muscle accretion, bone development, and fat catabolism.	➢ Blood	PCR	A significant association was observed between this SNP and several growth traits in which the genotype GG exhibited the best values.	China	[87]
Gansu	Tianzhu white yak/111 Qinghai Plateau yak/70 Xinjiang yak/50 Gannan yak/95 Datong yak/72	➢ Live weight; ➢ Average daily gain; ➢ Carcass weight; ➢ Viscera fat weight; ➢ Loin-eye area.	*LPL*	Lipoprotein lipase (*LPL*) is considered as a key enzyme in lipid deposition and metabolism in tissues. It is assumed to be a major candidate gene for genetic markers in lipid deposition.	Blood	PCR–SSCP analysis and DNA sequencing	The results indicate that the LPL gene is a strong candidate gene that affects carcass traits and fat deposition in yaks.	China	[88]

## Data Availability

All the data are provided in the text of the manuscript. There are no additional data sources.
